# Dr. Elliot's File Carrier

**Published:** 1848-10

**Authors:** 


*Dr. Elliot's File Carrier.*—We should have acknowledged our indebtedness to Dr. W. H. Elliot, of Montreal, long since, for a file carrier, invented by himself, which he had the kindness to send to us in the fall of 1847. It is so constructed that it may be used on either side of the mouth, and on either jaw, with equal facility. It is, like Dr. Westcott's, composed of a single piece of steel, and is so contrived that it will hold a flat, half round or V shaped file with equal facility, and so as to act upon a tooth in any desired direction. It has recently been modified by Mr. Chevalier, an instrument maker of New York, with a view of accommodating it to files of various lengths. The principle, however, is not changed in the least, nor do we regard the improvement as of any value whatever. In the absence of properly constructed files for separating the molar teeth, the instrument is of great value, and should be in the hands of every dentist. The dental profession is greatly indebted to Dr. Elliot for a number of very valuable instruments, invented by himself, a description of a number of which have already appeared in the Journal, as well as for many valuable contributions to the literature of practical dentistry, which have enriched the pages of this publication. These contributions have all been well received by our readers, and we hope he will continue them.—*Baltimore Ed.*

